# Editor’s Note

**DOI:** 10.15586/jkcvhl.2014.8

**Published:** 2014-04-22

Dear fellow researchers,

Welcome to the first issue of the Journal of Kidney Cancer and VHL (JKCVHL).

JKCVHL is a peer-reviewed, open access journal created to fill the vacuum for a specialized journal to disseminate information on the advances in kidney cancer and VHL research. I am confident JKCVHL will fulfil its intended purpose, and will be a leading journal in this field in the days to come.

